# Detection, measurement, and diagnosis of lung nodules by ultra-low-dose CT in lung cancer screening: a systematic review

**DOI:** 10.1093/bjro/tzae041

**Published:** 2024-11-22

**Authors:** Zhijie Pan, Yaping Zhang, Lu Zhang, Lingyun Wang, Keke Zhao, Qingyao Li, Ai Wang, Yanfei Hu, Xueqian Xie

**Affiliations:** Radiology Department, Shanghai General Hospital, Shanghai Jiao Tong University School of Medicine, Shanghai 200080, China; Radiology Department, Shanghai General Hospital, Shanghai Jiao Tong University School of Medicine, Shanghai 200080, China; Radiology Department, Shanghai General Hospital, Shanghai Jiao Tong University School of Medicine, Shanghai 200080, China; Radiology Department, Shanghai General Hospital, Shanghai Jiao Tong University School of Medicine, Shanghai 200080, China; Radiology Department, Shanghai General Hospital, Shanghai Jiao Tong University School of Medicine, Shanghai 200080, China; Radiology Department, Shanghai General Hospital, Shanghai Jiao Tong University School of Medicine, Shanghai 200080, China; Radiology Department, Shanghai General Hospital, University of Shanghai for Science and Technology, Shanghai 200093, China; Radiology Department, Shanghai General Hospital, Shanghai Jiao Tong University School of Medicine, Shanghai 200080, China; Radiology Department, Shanghai General Hospital, Shanghai Jiao Tong University School of Medicine, Shanghai 200080, China; Radiology Department, Shanghai General Hospital, Shanghai Jiao Tong University School of Medicine, Shanghai 200080, China

**Keywords:** lung nodule, ultra-low-dose CT, diagnostic test, systematic review

## Abstract

**Objective:**

There is a lack of recent meta-analyses and systematic reviews on the use of ultra-low-dose CT (ULDCT) for the detection, measurement, and diagnosis of lung nodules. This review aims to summarize the latest advances of ULDCT in these areas.

**Methods:**

A systematic review of studies in PubMed and Web of Science was conducted, using search terms specific to ULDCT and lung nodules. The included studies were published in the last 5 years (January 2019-August 2024). Two reviewers independently selected articles, extracted data, and assessed the risk of bias and concerns using the Quality Assessment of Diagnostic Accuracy Studies-II (QUADAS-II) tool. The standard-dose, low-dose, or contrast-enhanced CT served as the reference-standard CT to evaluate ULDCT.

**Results:**

The literature search yielded 15 high-quality articles on a total of 1889 patients, of which 10, 3, and 2 dealt with the detection, measurement, and diagnosis of lung nodules. QUADAS-II showed a generally low risk of bias. The mean radiation dose for ULDCT was 0.22 ± 0.10 mSv (7.7%) against 2.84 ± 1.80 mSv for reference-standard CT. Nodule detection rates ranged from 86.1% to 100%. The variability of diameter measurements ranged from 2.1% to 14.4% against contrast-enhanced CT and from 3.1% to 8.29% against standard CT. The diagnosis rate of malignant nodules ranged from 75% to 91%.

**Conclusions:**

ULDCT proves effective in detecting lung nodules while substantially reducing radiation exposure. However, the use of ULDCT for the measurement and diagnosis of lung nodules remains challenging and requires further research.

**Advances in knowledge:**

When ULDCT reduces radiation exposure to 7.7%, it detects lung nodules at a rate of 86.1%-100%, with a measurement variance of 2.1%-14.4% and a diagnostic accuracy for malignancy of 75%-91%, suggesting the potential for safe and effective lung cancer screening.

## Introduction

Lung cancer has a high mortality rate, with a five-year survival rate of 22.9%.^1^ CT screening can detect early-stage lung cancer and improve patient prognosis.^2^^,^^3^ Recent guidelines recommend continuous surveillance for patients with indeterminate nodules, but there is growing concern about cumulative radiation exposure.^4^^,^^5^

A reduction in radiation dose is often accompanied by an increase in image noise and a deterioration in image quality. Therefore, minimizing the radiation dose while maintaining image quality is highly valued.^6^ The radiation dose for a single chest scan is about 3-7 mSv for standard-dose CT (SDCT) and 1.5 mSv for low-dose CT (LDCT).^1^^,^^7^^,^^8^ The recent introduction of ultra-low-dose CT (ULDCT) has the potential to significantly reduce radiation exposure through advanced hardware and sophisticated image reconstruction algorithms.^8–11^ These developments highlight the potential of ULDCT in the safe detection and evaluation of lung nodules.^12^

Taekker et al^13^ analysed studies from 2002 to 2019 to provide a comprehensive systematic review on the diagnostic accuracy of LDCT and ULDCT for the detection of various chest pathologies, including lung nodules. However, there has been a lack of meta-analyses and systematic reviews on ULDCT in the last 5 years. Informed by the findings from Jiang et al^9^ and Carey et al,^11^ it is evident that the common ULDCT doses range from 0.13 to 0.49 mSv. Therefore, this study aims to provide a systematic review of the detection, measurement, and diagnosis of both solid and non-solid nodules in lung cancer screening using ULDCT with a radiation dose below 0.5 mSv, focusing on image reconstruction algorithms.

## Methods

This study adhered to the Preferred Reporting Items for Systematic Reviews and Meta-analyses.^14^

### Search strategy

A systematic literature search was conducted on PubMed and Web of Science for articles written in English using terms related to ULDCT and lung nodules. The reference lists of relevant articles and reviews were also screened for other possible studies. Table 1 shows the detailed search strategy.

**Table 1.** Literature searching strategy.

Searching terms used to identify relevant citations
PubMed (“tomography, x ray computed”[MeSH Terms] OR “CT”[Title/Abstract]) AND (“ultra low dose”[Title/Abstract] OR “ultra-low-dose”[Title/Abstract] OR “ultra low*”[Title/Abstract] OR “ultra-low*”[Title/Abstract]) AND (“neoplasms”[MeSH Terms] OR “neoplasm*”[Title/Abstract] OR “cancer”[Title/Abstract] OR “cancer*”[Title/Abstract] OR “tumor”[Title/Abstract] OR “tumor*”[Title/Abstract] OR “carcinoma”[Title/Abstract] OR “carcinoma*”[Title/Abstract] OR “mass”[Title/Abstract] OR “neoplastic”[Title/Abstract] OR (“solid lesion”[Title/Abstract] OR “solid lesion*”[Title/Abstract] OR “nodule”[Title/Abstract] OR “nodule*”[Title/Abstract]))
Web of Science TS = (“tomography, x ray computed” OR “CT”) AND TS = (“ultra low dose” OR “ultra-low-dose” OR “ultra low*” OR “ultra-low*”) AND TS = (“neoplasms” OR “neoplasm*” OR “cancer” OR “cancer*” OR “tumor” OR “tumor*” OR “carcinoma” OR “carcinoma*” OR “mass” OR “neoplastic” OR “solid lesion” OR “solid lesion*” OR “nodule” OR “nodule*”)

### Study selection criteria

After removing duplicate articles, 2 reviewers with 3 and 10 years’ experience in chest imaging assessed titles, abstracts, and full texts to select eligible articles. Inclusion criteria were as follows: (1) clinical studies in adult patients; (2) application of ULDCT in lung cancer screening; and (3) studies on the detection, measurement, and diagnosis of lung tumours or nodules. Exclusion criteria were: (1) patents, reviews, abstracts, case reports, letters, or conference proceedings; (2) effective radiation dose > 0.5 mSv from a single chest CT; (3) the primary purpose of CT scan was not for chest disease; (4) patient cohort size < 40 or no mention of patient cohort size. A sample size of 40 in the Student t-test provides a statistical power of > 0.8 at the 95% confidence level between the study and control groups, as estimated using the sample size analysis tool (MedCalc, MedCalc Software Ltd., Ostend, Belgium).

### Data extraction

The same 2 reviewers extracted information from the included articles, completed information extraction forms, and resolved discrepancies by consulting a radiologist with 20 years of experience. The extracted information included the name of the first author, year of publication, patient cohort size, scan information, clinical application, image reconstruction algorithm, and main findings.

The included articles were divided into 3 categories by clinical application: detection, measurement, and diagnosis of lung nodules. The image reconstruction algorithm refers to the algorithm used to generate ULDCT images, such as adaptive statistical iterative reconstruction-V (ASIR-V) and deep learning image reconstruction (DLIR). The main findings refer to the statistical metrics that show the performance of ULDCT, that is, detection rate of nodules, results of Bland-Altman analysis, absolute percent error (APE) for nodule measurements, and diagnosis performance of malignant nodules. The above analyses used SDCT, LDCT, or contrast-enhanced CT as the reference-standard CT, with one study also using ULDCT as the reference standard.^15^Table 2 presents the study characteristics and main findings of the included articles. Table 3 shows the sensitivity analysis of the included articles. 

**Table 2.** Study characteristic and main findings of the included articles.

Author name and publication year	Patient cohort size, *n*	Thickness, mm	Clinical application	Image reconstruction algorithm	Nodules size and type
Ludes et al. 2019^16^	51	0.5	Detection	AIDR-3D	Solid nodules > 5 mm
Gobi et al. 2022^17^	124	3	Detection	MBIR	Solid nodules > 3 mm
Gheysens et al. 2022^7^	63	NA	Detection	MBIR	Solid nodules > 50 mm^3^
Ye et al. 2021^18^	210	1.25	Detection	ASIR-V	Solid nodules > 1 mm pGGNs > 6 mm
Hata et al. 2020^19^^,^^a^	41	0.625	Detection	FBP, HIR, MBIR, HIR-DLD, MBIR-DLD	Solid nodules: 3 (1.6-26.3 mm) Part-solid nodules: 6.8 (2.9-26.3 mm) pGGNs: 5.1 (2.5-14.4 mm)
Miller et al. 2019^20^	99	5	Detection	FBP/ASIR, MBIR	Solid nodules > 4 mm
Meyer et al. 2019^15^^,^^b^	64	0.5	Detection	AIDR-3D	Solid nodules > 5 mm
Kerpel et al. 2021^21^	52	NA	Detection	ASIR-V	Solid nodules ≥ 4 mm
Yang et al. 2023^22^^,^^a^	147	0.625	Detection	FBP, HIR, DLIR	Solid nodules > 5 mm Part-solid nodules > 5 mm pGGNs > 5 mm
Gorenstein et al. 2023^23^^,^^a^	123	1.25	Detection	ASIR-V, DLIR	Solid nodules (1-15 mm) Part-solid nodules (1-5.5 mm) pGGNs (1-15 mm)
Zheng et al. 2024^24^^,^^a^	142	NA	Measurement	ASIR-V, DLIR	Solid nodules < 30 mm Part-solid nodules < 30 mm pGGNs < 30 mm
Zhao et al. 2022^10^	141	1.25	Measurement	FBP, ASIR-V, DLIR	Solid nodules (3-30 mm) Part-solid nodules (3-30 mm) pGGNs (3-30 mm)
Jiang et al. 2022^9^^,^^a^	203	1.25	Measurement	FBP, ASIR-V, DLIR	Solid nodules (3-30 mm) Part-solid nodules (3-30 mm)
Milanese et al. 2023^25^^,^^a^	361	1	Diagnosis	MBIR	Solid nodules (90-148 mm^3^) Part-solid nodules (93-262 mm^3^) pGGNs (93-262 mm^3^)
Autrusseau et al. 2021^26^	68	1	Diagnosis	AIDR-3D	Solid nodules (6-25 mm)

Author name and publication year	ULDCT dose, mSv	Reference standard dose, mSv	Main findings	
			Solid nodules	Non-solid nodules
Ludes et al. 2019^16^	ULDCT1:0.21 ULDCT2:0.22	SDCT: 3.1	Detection rate: 92.8%	NA
Gobi et al. 2022^17^	ULDCT: 0.50 ± 0.005	SDCT: 3.99 ± 1.57	Detection rate: 91%	NA
Gheysens et al. 2022^7^	ULDCT: 0.13	SDCT: 1.7	Detection rate: 86.1%	NA
Ye et al. 2021^18^	ULDCT: 0.16 ± 0.03	LDCT: 0.80 ± 0.28	Detection rate: 96.6%	Detection rate: 93%
Hata et al. 2020^19^^,^^a^	ULDCT: 0.19 ± 0.01	SDCT: 6.46 ± 2.28	Detection rate: 87.5%-100%	
Miller et al. 2019^20^	ULDCT: 0.13	LDCT: 1.67	Detection rate: 98.5%	NA
Meyer et al. 2019^15^^,^^b^	ULDCT WV: 0.20 ± 0.02	ULDCT helical: 0.22 ± 0.02	Detection rate: 95.5%	NA
Kerpel et al. 2021^21^	ULDCT: 0.23 ± 0.08	SDCT: 6.39 ± 2.5	Detection rate: 93%-96%	NA
Yang et al. 2023^22^^,^^a^	ULDCT: 0.18	SDCT: 3.28	Detection rate: 94.7%	
Gorenstein et al. 2023^23^^,^^a^	ULDCT: 0.24 ± 0.08	LDCT: 1.01 ± 0.06	Detection rate: 98.1%	
Zheng et al. 2024^24^^,^^a^	ULDCT1:0.57 ± 0.04 ULDCT2:0.33 ± 0.03	SDCT: 4.32 ± 0.33	APE: 3.10% ± 95.11%	
Zhao et al. 2022^10^	ULDCT1:0.07 ULDCT2:0.14	CECT: 2.38	Bland-Altman analysis: 2.3% (1.6%-3.0%)	Bland-Altman analysis: 2.1% (0.4%-3.9%)
Jiang et al. 2022^9^^,^^a^	ULDCT1:0.07 ULDCT2:0.14	CECT: 2.38 ± 0.37	Bland-Altman analysis: 14.4% (10.6%-18.2%)	
Milanese et al. 2023^25^^,^^a^	ULDCT: 0.31, 0.36, 0.27 and 0.37	LDCT: 1.62	Diagnosis rate: 88%-91%	
Autrusseau et al. 2021^26^	ULDCT: 0.23 ± 0.02	SDCT: 3.3 ± 1.9	Diagnosis rate: 75%	NA

a Hata et al. 2020,^19^ Yang et al. 2023,^22^ Gorenstein et al. 2023,^23^ Zheng et al. 2024,^24^ Jiang et al. 2022,^9^ and Milanese et al. 2023^25^ put together the results for solid and non-solid nodules.

b Meyer et al. 2019^15^ is a comparison of the ability of two different modalities of ULDCT to detect nodules (helical vs wide-volume).

Abbreviations: AIDR-3D = adaptive iterative dose reduction-3D; ASIR-V = adaptive statistical iterative reconstructions-V; CECT = contrast-enhanced CT; DLD = deep learning-based denoising; DLIR = deep learning image reconstruction; FBP = filtered back projection; HIR = hybrid iterative reconstruction; LDCT = low-dose CT; MBIR = model-based iterative reconstruction; NA = not available; SDCT = standard-dose CT; ULDCT = ultra-low-dose CT; WV = wide-volume.

**Table 3.** Sensitivity and specificity analysis of the included articles.

Author name and publication year	Solid nodules						Non-solid nodules					
	Sensitivity	Specificity	LR+	LR-	DOR	AUC	Sensitivity	Specificity	LR+	LR-	DOR	AUC
Ludes et al. 2019^16^	92.8%	NA					NA					
Gobi et al. 2022^17^	91%	100%	∞	0.09	∞		NA					
Gheysens et al. 2022^7^	86.1%	71.4%	3.01	0.19	15.84		NA					
Ye et al. 2021^18^^,^^a^	96.6%	100%	∞	0.03	∞	0.75	93%	NA				0.72
Hata et al. 2020^19^^,^^a^	87.5%-100%	84.5%-95.9%	9.57	0.07	138.7	0.92	87.5%-100%	84.5%-95.9%	9.57	0.07	138.7	0.92
Miller et al. 2019^20^	98.5%	100%	∞	0.02	∞		NA					
Meyer et al. 2019^15^	95.5%	97%	31.83	0.05	691		NA					
Kerpel et al. 2021^21^	93%-96%	88%-100%	15.75	0.06	264		93%-96%	88%-100%	15.75	0.06	264	
Yang et al. 2023^22^	94.7%	NA					94.7%	NA				
Gorenstein et al. 2023^23^	98.1%	91.7%	11.82	0.02	562.9		98.1%	91.7%	11.82	0.02	562.9	
Zheng et al. 2024^24^	99.11%	NA					88.28%-100%	NA				
Zhao et al. 2022^10^	NA						NA					
Jiang et al. 2022^9^	85.5%	NA					NA					
Milanese et al. 2023^25^	88%-91%	NA					88%-91%	NA				
Autrusseau et al. 2021^26^	75%	85%	5	0.29	17.24		NA					

Author name and publication year	Solid nodules				Non-solid nodules			
	TP	FP	FN	TN	TP	FP	FN	TN
Ludes et al. 2019^16^	NA				NA			
Gobi et al. 2022^17^	22	0	2	2	NA			
Gheysens et al. 2022^7^	118	4	19	10	NA			
Ye et al. 2021^18^^,^^a^	228	0	8	135	93	12	28	237
Hata et al. 2020^19^^,^^a^	119	1	20	113	119	1	20	113
Miller et al. 2019^20^	179	0	3	20	NA			
Meyer et al. 2019^15^	15	1	1	15	NA			
Kerpel et al. 2021^21^	NA				NA			
Yang et al. 2023^22^	NA				NA			
Gorenstein et al. 2023^23^	62	5	1	57	NA			
Zheng et al. 2024^24^	NA				NA			
Zhao et al. 2022^10^	NA				NA			
Jiang et al. 2022^9^	NA				NA			
Milanese et al. 2023^25^	NA				NA			
Autrusseau et al. 2021^26^	9	13	3	74	NA			

a Only Ye et al. 2021^18^ and Hata et al. 2020^19^ provide the values for AUC.

Ludes et al. 2019,^16^ Zheng et al. 2024,^24^ Yang et al. 2023,^22^ Zhao et al. 2022,^10^ Jiang et al. 2022,^9^ Milanese et al. 2023^25^ lack complete data on sensitivity and specificity. Kerpel et al. 2021^21^ lack sample number of nodules. Only Gheysens et al. 2022,^7^ Ye et al. 2021^18^ and Autrusseau et al. 2021^26^ provide the values for TP, FP, FN, and TN. The values of TP, FP, FN, and TN in Gobi et al. 2022,^17^ Hata et al. 2020,^19^ Miller et al. 2019,^20^ Meyer et al. 2019,^15^ and Gorenstein et al. 2023^23^ are obtained through calculations.

### Study quality assessment

The risk of bias and concerns regarding the applicability of the included studies were assessed using the Quality Assessment of Diagnostic Accuracy Studies-II (QUADAS-II) tool.^27^ This tool consists of 2 sections: the first addresses issues related to subject selection and reference setting, while the second addresses issues related to subject utility and reference standard.

In this review, the risk of bias was assessed in 4 areas: patient selection, index test, reference standard, and flow and timing. Patient selection refers to whether patients undergoing ULDCT were sampled consecutively or randomly. Index test refers to whether the ULDCT results are interpreted while blinded to the reference-standard CT results. Reference standard answers whether the reference-standard CT results effectively classify the target condition. Flow and timing domain addresses the adequacy of the interval between the ULDCT and the reference-standard CT. Similarly, the concerns regarding the applicability were assessed from three perspectives: patient selection, index test, and reference standard. Each item was scored on a 3-point scale (high, unclear, and low).^28^ The same 2 radiologists performed the QUADAS-II assessment and resolved discrepancies by consultation with the third senior radiologist.

### Sensitivity analysis

To assess the robustness of the results, a two-step sensitivity analysis was performed: (1) reanalysis of the data excluding studies identified as having a high risk of bias to evaluate their impact on the overall pooled estimate; and (2) sequential omission of individual studies to assess the influence of each study on the pooled estimates.

## Results

### Literature selection

An initial screening identified 345 citations in the last 5 years (January 2019-August 2024), of which 303 were excluded by title and abstract. The excluded citations included 32 non-original articles, 62 non-clinical studies, 58 not associated with nodules or masses, 102 non-CT examinations, and 49 non-ULDCT.

**Figure 1.**
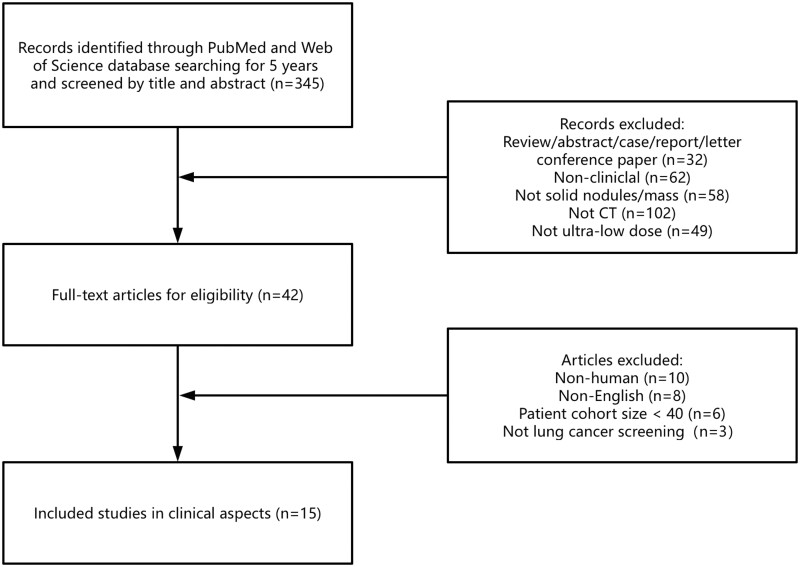
Literature review and selection flowchart.

After reading the full texts of the remaining 42 articles, 27 were further excluded, of which 10 were not on patients, 8 not in English, 6 with a patient cohort size <40, and 3 not associated with lung cancer screening. Finally, 15 articles on a total of 1889 patients were included in this systematic review. Figure 1 shows the flowchart of the literature search and selection.

### Literature characteristics

Of the 15 included articles, 10 focused on nodule detection, 3 on measurement,^9^^,^^10^^,^^24^ and 2 on diagnosis.^25^^,^^26^ With regard to nodule type, all 15 studies investigated solid nodules, 7 examined part-solid nodules,^9^^,^^10^^,^^19^^,^^22^^,^^23^^,^^24^^,^^25^ and 2 examined 7 ground-glass nodules (pGGN).^10^^,^^18^^,^^19^^,^^22^^,^^23^^,^^24^^,^^25^

### Reconstruction algorithms

Of the 15 articles, the implementation of ULDCT was based on several image reconstruction algorithms, including adaptive iterative dose reduction-3D (AIDR-3D) in 3 articles,^15^^,^^16^^,^^26^ model-based iterative reconstruction (MBIR) in 5 articles,^7^^,^^17^^,^^19^^,^^20^^,^^25^ ASIR-V in 6 articles,^9^^,^^10^^,^^18^^,^^21^^,^^23^^,^^24^ and DLIR in 5 articles.^9^^,^^10^^,^^22^^,^^23^^,^^24^ The mean effective radiation dose of ULDCT for the patients in all included studies was 0.22 ± 0.10 mSv (range from 0.07 to 0.50 mSv).

Several image reconstruction algorithms were used in the reference-standard CT, including AIDR-3D in three articles,^15^^,^^16^^,^^26^ MBIR in 3 articles,^7^^,^^17^^,^^25^ ASIR-V in 5 articles,^18^^,^^20^^,^^21^^,^^23^^,^^24^ and filtered back projection (FBP) in 4 articles.^9^^,^^10^^,^^19^^,^^22^ The mean radiation dose of the reference-standard CT was 2.84 ± 1.80 mSv (range from 0.22 to 6.46 mSv).

### Literature quality

**Figure 2.**
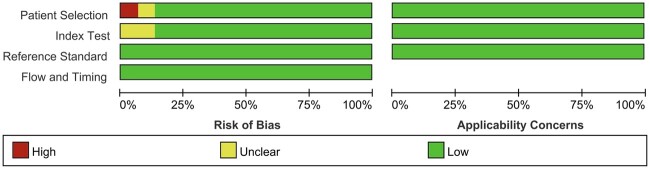
Assessment of individual risk of bias domains and concerns regarding applicability.

**Figure 3.**
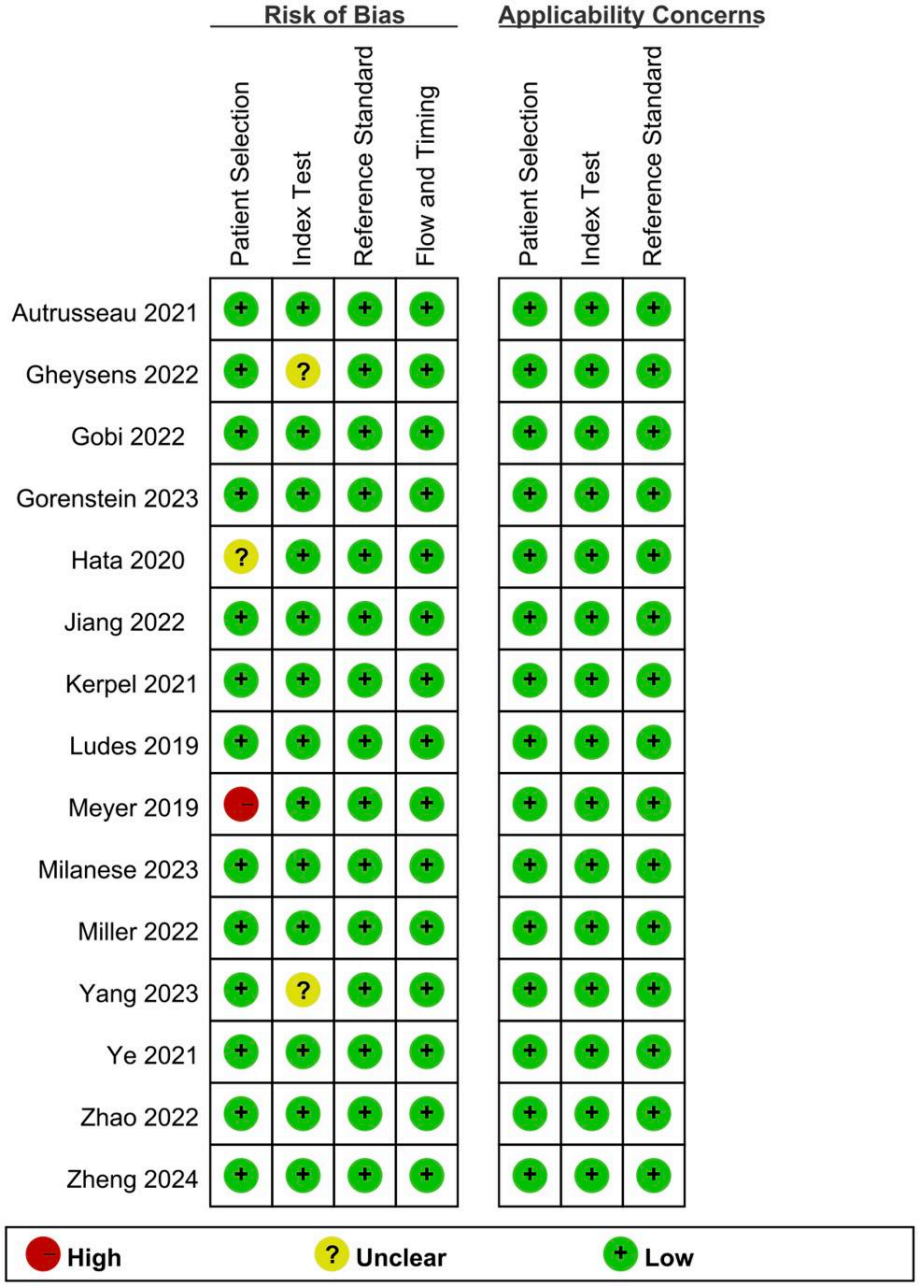
Assessment of risk of bias and concerns regarding applicability according to the QUADAS-II tool.

Figures 2 and 3 illustrate the results of the risk of bias and concerns regarding the applicability, as evaluated by the QUADAS-II tool. In terms of the risk of bias, all 15 studies were rated as low risk for the reference standard, flow and timing. For patient selection, 13 (87%) were rated as low risk, one (7%) was rated as unclear due to the lack of reporting on the patient selection process,^19^ while one (7%) was rated as high risk due to the absence of a consecutive or random sample of patients.^15^ For the index test, 13 studies (87%) were rated as low risk, while two (13%) were rated as unclear.^7^^,^^22^ With respect to concerns of applicability, all 15 studies were rated as low concern for patient selection, index test, and reference standard.

### Nodule detection

**Figure 4.**
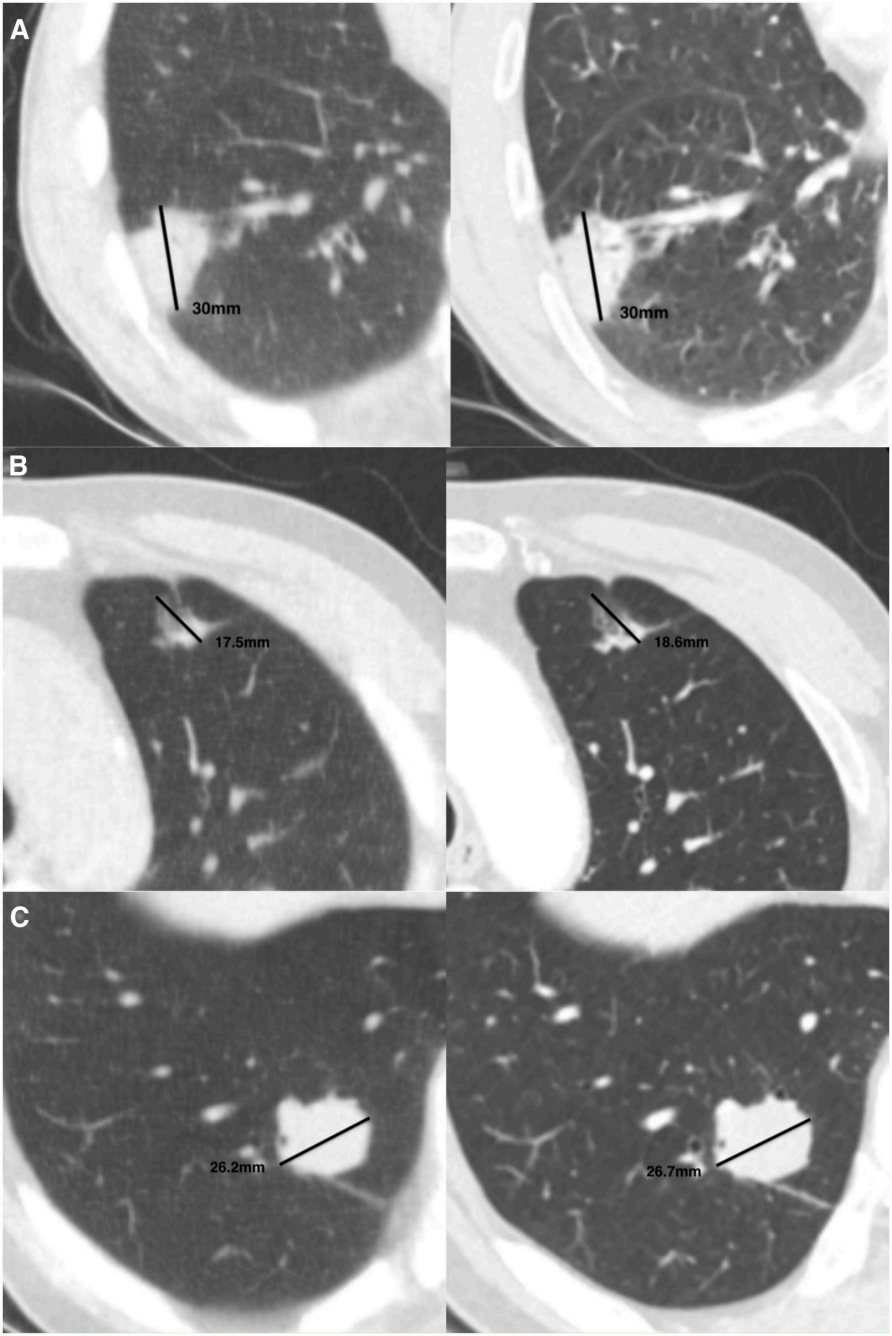
Representative cases. Comparison of ULDCT images (left) and diagnostic contrast-enhanced CT images (right). The first two ULDCT scans (A and B) had a radiation dose of 0.07 mSv (70 kV/20 mA). The third ULDCT scan (C) had a radiation dose of 0.14 mSv (70 kV/40 mA). The mean radiation dose of the contrast-enhanced CT scans (A, B, and C) is 2.24 mSv (120 kV/100-200 mA). The dose of ULDCT is only 3%-6% of that of contrast-enhanced CT. (A) A 64-year-old male patient with a 30-mm lobulated nodule in the basal segment of the lower lobe of the right lung, as detected and measured by ULDCT and contrast-enhanced CT, which was pathologically confirmed as invasive squamous carcinoma. (B) A 56-year-old male patient with chronic inflammation of the bronchial mucosa detected by biopsy, had a part-solid nodule in the apical posterior segment of the upper lobe of the left lung measuring 17.5 mm by ULDCT and 18.6 mm by contrast-enhanced CT. The morphology of this nodule is irregular with bronchogram. (C) A 57-year-old man with a lobulated nodule in the basal segment of the lower lobe of the right lung, measuring 26.2 mm by ULDCT and 26.7 mm by contrast-enhanced CT, was pathologically confirmed as invasive adenocarcinoma. Although the malignant nodules detected by ULDCT appear to be ill-defined compared with diagnostic contrast-enhanced CT, the shape of these nodules could be recognized and their size could be measured. (These cases are from the author's institute.)

Compared with reference-standard CT, ULDCT showed a high detection rate for both solid and non-solid nodules.^18^,^19^,^22^,^23^ Of the 10 included studies on nodule detection, the detection rate ranged from 86.1% to 100% for solid nodules, and 87.5% to 100% for non-solid nodules. The lowest detection rate, reported by Gheysens et al,^7^ was for nodules > 50 mm³ in a cohort of 63 patients. Notably, ULDCT achieved a 100% detection rate for nodules classified as Lung-RADS category 3 or above,^19^ providing confidence in the detection of suspected malignant nodules. Figure 4 illustrates some representative cases of patients diagnosed with malignant nodules after undergoing ULDCT and contrast-enhanced CT.

### Nodule measurement

Two studies evaluated nodule measurement using contrast-enhanced CT as the reference standard^9^^,^^10^ and 1 study used SDCT.^24^ Zhao et al^10^ studied the measurement discrepancy of nodules > 10 mm in diameter and observed a discrepancy of 2.3% and 2.1% for solid and non-solid nodules, respectively. Jiang et al^9^ investigated the measurement discrepancy of solid and non-solid nodules from 3 to 30 mm in diameter, and the Bland-Altman analysis showed a discrepancy of 14.4% between ULDCT and contrast-enhanced CT. Zheng et al^24^ compared the APE of lung nodule volume measurements between DLIR-reconstructed ULDCT images and ASIR-V-reconstructed contrast-enhanced CT images. ULDCT showed a significantly lower APE (3.10% ± 95.11% vs 8.29% ± 99.14%, *P* < .001).

### Nodule diagnosis

Autrusseau et al^26^ investigated the diagnosis of seven malignant nodules using ULDCT through radiomics analysis. Their results showed a diagnostic accuracy of 94%, comparable to that of SDCT. Milanese et al^25^ evaluated 18 malignant nodules for classification concordance between ULDCT and LDCT based on LungRADS. The results showed high concordance and a diagnostic accuracy of 75% for ULDCT. Despite the limited sample size, these studies tentatively suggest that ULDCT can be used to diagnose malignant lung nodules.

### Sensitivity analysis

For solid nodules, the sensitivity ranged from 75% to 100%, the specificity from 71.4% to 100%, the positive likelihood ratio (LR+) was above 3.01, the negative likelihood ratio (LR-) ranged from 0.02 to 0.19, and the diagnostic odds ratio (DOR) was above 17.24. Only Ye et al^18^ and Hata et al^19^ reported AUC values of 0.75 and 0.92, respectively. For non-solid nodules, the sensitivity ranged from 87.5% to 100%, the specificity from 84.5% to 100%, LR+ ranged from 9.57 to 15.75, LR- from 0.02 to 0.07, and DOR from 138.7 to 562.9. Ye et al^18^ and Hata et al^19^ reported AUC values of 0.72 and 0.92, respectively.

**Figure 5.**
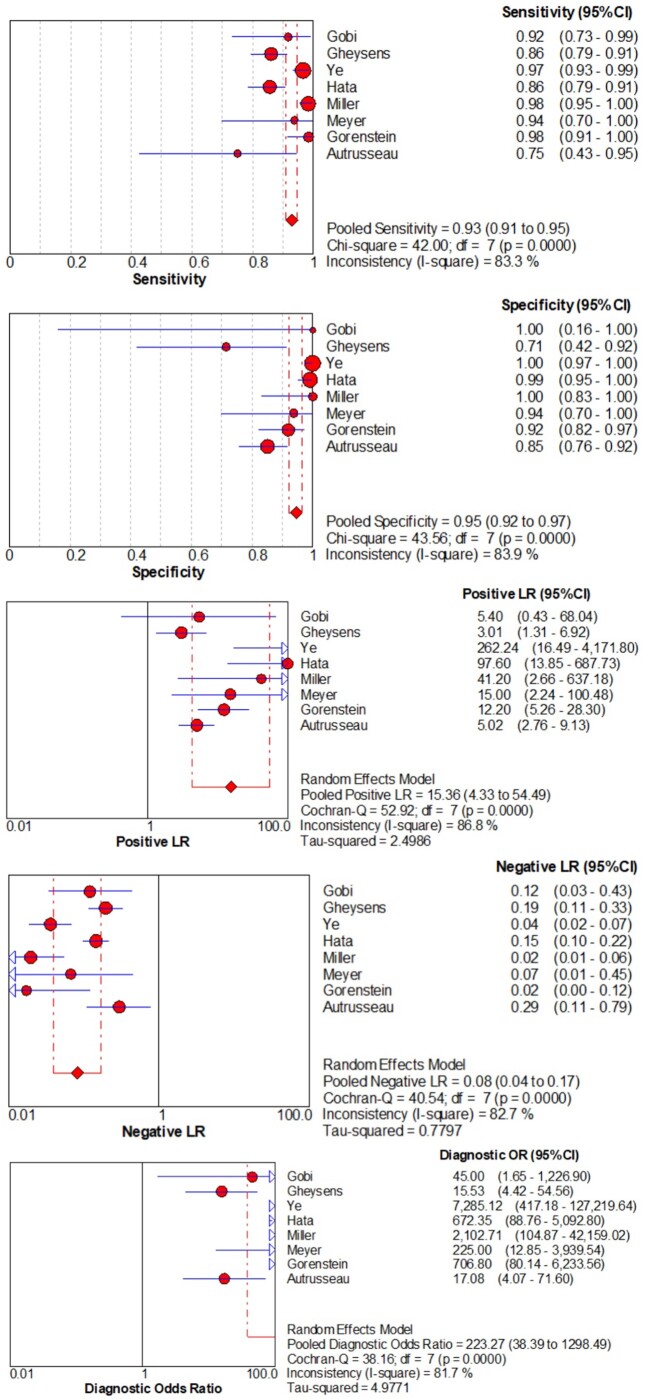
Forest plot of sensitivity, specificity, positive likelihood ratio (LR+), negative likelihood ratio (LR-), and DOR for included studies.

Figure 5 illustrates the forest plot for a subset of the included literature. Sensitivity analysis was conducted to explore the effect of individual studies on overall heterogeneity (*I*^2^). The baseline *I*^2^, including all 8 studies^7^^,^^15^^,^^17–20^^,^^23^^,^^26^ (Table 3), was 83.3%, suggesting substantial heterogeneity. After excluding the study with a high risk of bias (Meyer et al^15^), the change in heterogeneity was minimal, that is, the *I*^2^ increased slightly to 85.7%. After successive exclusion of other individual studies, the *I*^2^ values ranged from 78.8% to 85.7%. Notably, the lowest *I*^2^ value (78.8%) was found after the exclusion of the study by Miller et al,^20^ whereas a slightly lower *I*^2^ of 81.3% was found after the exclusion of the study by Gheysens et al.^7^

## Discussion

Advances in CT technology, particularly in image reconstruction algorithms, have contributed significantly to the evolution of ULDCT, allowing the detection, measurement, and diagnosis of lung nodules with markedly reduced radiation exposure. The included articles in this review show that ULDCT, with an average radiation dose of 0.22 mSv, can detect lung nodules using only 7.7% of the radiation dose of the reference-standard CT (2.84 mSv), suggesting that advances in algorithms have made it possible to maintain image quality even at low dose scans.

Iterative reconstruction optimizes an objective function to reconstruct images. Leon et al^29^ reported that AIDR-3D can reduce the dose while maintaining image quality compared with FBP. Han et al^30^ and Lee et al^31^ reported that ASiR-V reduced image noise while improving signal-to-noise and contrast-to-noise ratios. Yamada et al^32^ and Godic et al^33^ reported that MBIR can model X-ray generation, tissue attenuation, and noise sources to further reduce radiation dose. The DLIR algorithm, trained on FBP-reconstructed images from SDCT scans as ground truth, has been optimized to preserve anatomical details while denoising the output images.^34^ Furthermore, DLIR has improved signal-to-noise and contrast-to-noise ratios, while reducing dose with minimal texture changes.^35^

ULDCT is an increasingly popular tool for the detection of lung nodules. Miller et al^20^ showed that ULDCT can reliably detect lung nodules >4 mm at a radiation dose comparable to that of radiography. Gobi et al^17^ reported a detection rate of > 90% for solid nodules >3 mm. Regarding nodule size and detection rate, Gheysens et al^7^ reported that scoutless ULDCT had a reliable detection rate for solid nodules >50 mm^3^, but a low detection rate for nodules < 50 mm^3^. Based on Lung-RADS classification, ULDCT detected 100% of solid nodules >6 mm and 98.1% of solid nodules >1 mm.^18^^,^^23^ However, the detection of pGGN and part-solid nodules by ULDCT is challenging due to their low contrast and unclear structure.^19^ Ye et al^18^ reported that ULDCT detected 93% of pGGN > 6 mm, whereas Yang et al^22^ found that ULDCT detected 91.1% of pGGN > 5 mm and 100% of part-solid nodules > 5 mm. Notably, deep learning-based techniques have emerged in image processing, that have proven to be valuable in improving image quality. For example, Hata et al^19^ demonstrated the efficacy of combining ULDCT scanning with deep learning-based denoising in improving image quality and optimizing Lung-RADS classification. Yang et al^22^ demonstrated that ULDCT combined with DLIR and computer-assisted diagnosis (CAD) is a viable alternative to SDCT protocols. Similarly, Ma et al^36^ found that ULDCT with DLIR reconstruction produced results comparable to SDCT in quantitative CAD analysis and image quality assessment of lung nodules. Taekker et al^13^ reviewed the literature from 2002 to 2019 and reported that the detection rate of lung nodules by ULDCT ranged from 85% to 96%. However, due to the varying chest conditions of the included patients, Taekker’s review could not determine whether ULDCT is consistently suitable for lung nodule detection. Although the detection rates in our study, ranging from 86.1% to 100%, show little change from the previous review, in recent years an increasing number of studies have supported the use of ULDCT to detect lung nodules. With technological advances, ULDCT has the potential for more widespread use in lung cancer screening.

Traditionally, lung nodules are measured manually to determine long- and short-axis diameters.^37^ However, some researchers have proposed that volumetric measurements are more reliable, given that the geometry of lung nodules is rarely perfectly spherical.^38^^,^^39^ Nevertheless, different algorithms may affect the results of nodule volume measurements.^40^^,^^41^ Although contrast-enhanced CT is commonly used to measure lung nodules and assess tumour response to therapy, it produces a high radiation dose.^42^ Recent experiments have shown that low-dose settings combined with deep learning-based algorithms can improve image quality while reducing radiation exposure.^34^^,^^43^ Bland-Altman analysis by Jiang et al^9^ showed a variation of 6.2% in diameter and 14.4% in volume of solid nodules, between DLIR-reconstructed ULDCT images and contrast-enhanced CT images, and the variations were 1.1% and 0.9% in non-solid nodules, respectively. Due to partial volume effects, DLIR slightly underestimated the long diameter and volume of non-solid nodules, while overestimating solid nodules. Kerpel et al^21^ concluded that the use of deep-learning techniques can reduce image noise on any CT scanner without the need for additional physical filters or hardware.

In the diagnosis of lung nodules, CT imaging and textural features are often used to determine the malignancy of nodules and to predict overall survival.^26^^,^^44^ The difficulty lies in identifying benign nodules that do not require follow-up and malignant nodules that require treatment. Autrusseau et al^26^ studied the diagnosis of seven malignant nodules using ULDCT by radiomics analysis and found that the accuracy of ULDCT in diagnosing malignant nodules was comparable to that of SDCT. Milanese et al^25^ compared the ability of ULDCT and LDCT to classify 18 malignant nodules according to LungRADS and found excellent agreement. Despite the limited sample size, these studies tentatively suggest that ULDCT can be used to diagnose malignant lung nodules. However, further studies with larger sample sizes are necessary to confirm these findings.

This systematic review confirmed the high overall sensitivity and specificity of ULDCT in detecting lung nodules. The pooled sensitivity and specificity were 93% and 95%, respectively, suggesting strong diagnostic performance across studies. The high sensitivity suggests that ULDCT is highly effective in the early detection of lung nodules, particularly in high-risk populations. The high specificity further supports its utility in accurately ruling out benign cases, thereby reducing the likelihood of false positives. The pooled LR+ was 15.36, indicating that a positive ULDCT result substantially increases the probability of a true positive diagnosis, while the LR- was 0.08, indicating that a negative result significantly decreases the likelihood of disease. The combined DOR was 223.27, highlighting the overall diagnostic strength of ULDCT. The observed heterogeneity suggests variability among the studies, possibly due to differences in patient populations, imaging protocols, or technical factors. Subgroup analyses revealed that studies using the DLIR algorithm exhibited more consistent sensitivity and specificity results. The AUC of the studies in this review ranged from 0.72 to 0.92, indicating moderate to high diagnostic accuracy of ULDCT in detecting lung nodules. The diagnostic accuracy was particularly high for nodules >5 mm. This is important in the clinical setting because larger nodules require differential diagnosis and therefore clearer imaging. In contrast, the detection of smaller nodules (<5 mm) or non-solid nodules is more challenging as these nodules are often benign or indeterminate and less clinically urgent. The diagnostic performance of different nodule types varies, with higher sensitivity for solid nodules and greater variability in specificity for non-solid nodules, highlighting the difficulty in accurately detecting smaller or less dense lesions. Although ULDCT has demonstrated significant potential as a reliable tool for the detection of lung nodules, further optimization is needed to improve the ability to detect smaller or non-solid nodules.

In sensitivity analyses, the original meta-analysis included all eight studies,^7^^,^^15^^,^^17–20^^,^^23^^,^^26^ with an *I*^2^ of 83.3%. When the study with a high risk of bias^15^ was excluded, the *I*^2^ increased slightly to 85.7%, suggesting a minimal effect on heterogeneity. After excluding other studies in turn, the *I*^2^ values ranged from 78.8% to 85.7%, indicating a small effect on heterogeneity. Notably, the lowest *I*^2^ value (78.8%) was found after excluding the study by Miller et al,^20^ whereas the *I*^2^ value decreased to 81.3% after excluding the study by Gheysens et al.^7^ These results suggest that none of the studies had a significant impact on the overall heterogeneity, supporting the robustness of the pooled results, despite the heterogeneity across studies.

This systematic review has several limitations. First, the methodological heterogeneity of the included studies in each evaluation section limits the possibility of pooling effect sizes in meta-analyses. Second, of the 15 included articles, only 3 were on nodule measurements and 2 were on diagnosis. Therefore, it may not provide a comprehensive picture of the overall situation, and we expect more articles on ULDCT. Third, in the included literature, the paucity of data in some studies may potentially affect the results of the meta-analysis. Fourth, although we included studies from the last 5 years, reflecting recent advances in ULDCT technology, relevant literature from 5 years ago is also important for understanding the development of ULDCT.^45–50^

### Prospects

In addition to the algorithms cited in this review, newly developed algorithms may also benefit ULDCT. For instance, the Precise IQ Engine, a super-resolution reconstruction algorithm, has shown potential to improve the outputs of ultra-high-resolution CT, surpassing traditional models in diagnostic accuracy for in-stent restenosis.^51^ In a phantom study, the latest deep learning-based image reconstruction algorithms demonstrated superior volumetric measurement accuracy for solid and non-solid nodules.^52^

The advent of photon-counting detector CT (PCD-CT) represents a major breakthrough in medical imaging, offering higher resolution and contrast at lower radiation doses than conventional scanners.^53^ Several studies have used PCD-CT to identify lung nodules, with promising results.^54–57^

## Conclusion

The advances in CT technology, particularly in image reconstruction algorithms, have facilitated the development of ULDCT to detect lung nodules at a significantly reduced radiation dose. This makes ULDCT a valuable tool for reducing the risk of radiation exposure in lung cancer screening, potentially allowing for broader screening initiatives and earlier detection. Nevertheless, further clinical validation and refinement of low-dose techniques are necessary to optimize ULDCT for lung cancer screening.
